# Robust mouse tracking in complex environments using neural networks

**DOI:** 10.1038/s42003-019-0362-1

**Published:** 2019-03-29

**Authors:** Brian Q. Geuther, Sean P. Deats, Kai J. Fox, Steve A. Murray, Robert E. Braun, Jacqueline K. White, Elissa J. Chesler, Cathleen M. Lutz, Vivek Kumar

**Affiliations:** The Jackson Laboratory, 600 Main Street, Bar Harbor, ME 04609 USA

## Abstract

The ability to track animals accurately is critical for behavioral experiments. For video-based assays, this is often accomplished by manipulating environmental conditions to increase contrast between the animal and the background in order to achieve proper foreground/background detection (segmentation). Modifying environmental conditions for experimental scalability opposes ethological relevance. The biobehavioral research community needs methods to monitor behaviors over long periods of time, under dynamic environmental conditions, and in animals that are genetically and behaviorally heterogeneous. To address this need, we applied a state-of-the-art neural network-based tracker for single mice. We compare three different neural network architectures across visually diverse mice and different environmental conditions. We find that an encoder-decoder segmentation neural network achieves high accuracy and speed with minimal training data. Furthermore, we provide a labeling interface, labeled training data, tuned hyperparameters, and a pretrained network for the behavior and neuroscience communities.

## Abstract (web summary)

Brian Geuther et al. use machine vison techniques to develop a mouse tracker based on an encoder-decoder segmentation neural network that is highly accurate even with minimal training data. This tracker can be adapted to various behavioral paradigms and animal models widely used for biomedical research.

## Introduction

Behavior is primarily an output of the nervous system in response to internal or external stimuli. It is hierarchical, dynamic, and high dimensional, and is generally simplified for analysis^1,2^. For instance, the rich locomotor movement performed by a mouse that is captured in video is routinely abstracted to either a simple point, a center of mass, or an ellipse for analysis. In order to track mice well with current methods, the experimental environment is simplified to obtain optimal contrast between the mouse and background for proper segmentation. Segmentation, a form of background subtraction, classifies pixels belonging to mice from background in video and enables these high level abstractions to be mathematically calculated. During mouse behavioral assays, the arena background color is often changed depending on the animal’s coat color, potentially altering the behavior itself^3–5^. Current trends in tracking include a wide variety of challenges to overcome^5^, including robustness to environmental complexity, robustness to subject diversity, tracking multiple subjects in the same environment, and preserving identities for multiple subjects. While much of recent progress has been made on solving the problem of multiple subject tracking^6–8^, little progress has been made on improving robustness to environmental complexity and subject diversity^9^. Making changes to the environment or subject appearance comes at a development cost, as current video tracking technologies cannot be applied in complex and dynamic environments or with genetically heterogeneous animals without a high level of user involvement, making both long-term experiements and large experiments unfeasible.

We sought to overcome these difficulties by building a robust next-generation mouse tracker that uses neural networks and achieves high performance under complex and dynamic environmental conditions, is indifferent to coat color, and does not require persistent supervision by the user. Convolutional neural networks are computational models that are composed of multiple spatial processing layers that learn representations of data with multiple levels of abstraction. These methods have dramatically improved the state-of-the-art in speech recognition, visual object recognition, object detection, and many other domains such as drug discovery and genomics^10^. Neural networks have a variety of possible applications for solving laboratory animal-tracking problems including improving segmentation quality, regressing posture parameters, predicting keypoint locations, and preserving identities over time. Semantic segmentation, a problem recently solved using deep learning, provides generalization power to dynamic environments where traditional background subtraction approaches would fail. While many segmentation structures are available each with their own set of advantages^11^, the most notable networks are FCN^12^, SegNet^13^, and U-Net^14^. Posture parameter regression is often realized by region-proposal networks, notably solved by Faster rcnn^15^. Instance segmentation networks, which join the task of localization and segmentation, include Mask r-cnn^16^, FCIS^17^, and Deep Learned Instance Metrics^18^. Keypoint predictions, commonly applied to the human pose estimation problem, allows tracking of individual keypoints. The converged architecture for keypoint tracking is a stacked hourglass^19^, most recently implemented by DeepCut^20^ and DeepLabCut^21^. Identity preservation is an ever-growing field for applications of neural networks, ranging from human face verification networks^22,23^ to animal identity recognition^6,7,24^. One of the key advantages of neural networks is that once an efficient network with suitable hyperparameters has been developed, it can easily be extended to other tasks by simply adding appropriate training data^25^. Application of these approaches is often limited as approaches often require annotated datasets with millions of annotated examples to function. Thus, we sought to build a highly generalizable solution for mouse tracking.

Current technologies restrict experimental paradigms from tracking diverse mouse populations, such as the diversity outbred mouse panel^26^, due to poor performance on subject diversity. As neuroscience and behavior moves into an era of big behavioral data^2^ and computational ethology^27^, improved methods are necessary that enable tracking animals in semi-natural and dynamic environments over long periods of time. To address this shortfall, we developed a robust scalable method of mouse tracking in an open field using modern convolutional neural network architecture. Our trained neural network is capable of tracking all commonly used strains of mice—including mice with different coat colors, body shapes, and behaviors—under multiple experimental conditions using a simple learn by example approach. Thus we present a scalable and robust solution that allows tracking in diverse experimental conditions.

## Results

### Traditional single mouse tracking approaches

We first used existing tracking methods to track 58 different mouse strains in multiple environments, and found them inadequate for our large-scale strain survey experiment (1845 videos, 1691 h). We tracked all the videos in this experiment using both Ctrax^28^, a modern open-source tracking software package that uses background subtraction and blob detection heuristics, and LimeLight (Actimetrics, Wilmette, IL), a commercially available tracking software package that uses a proprietary tracking algorithm. Ctrax uses a background subtraction algorithm to abstract a mouse on a per frame basis to five metrics: major and minor axis, *x* and *y* location of center of the mouse, and the direction of the animal^28^. LimeLight uses a single key-frame background model for segmentation and detection, abstracting the mouse to a center of mass using a proprietary algorithm. Other available tracking software packages includes CADABRA^29^, EthoVision^30^, idTracker^31^, idTracker.ai^6^, ToxTrac^8^, MiceProfiler^32^, MOTR^33^, Cleversys TopScan (http://cleversysinc.com/CleverSysInc/), Autotyping^34^, and Automated Rodent Tracker^35^.

**Fig. 1:**
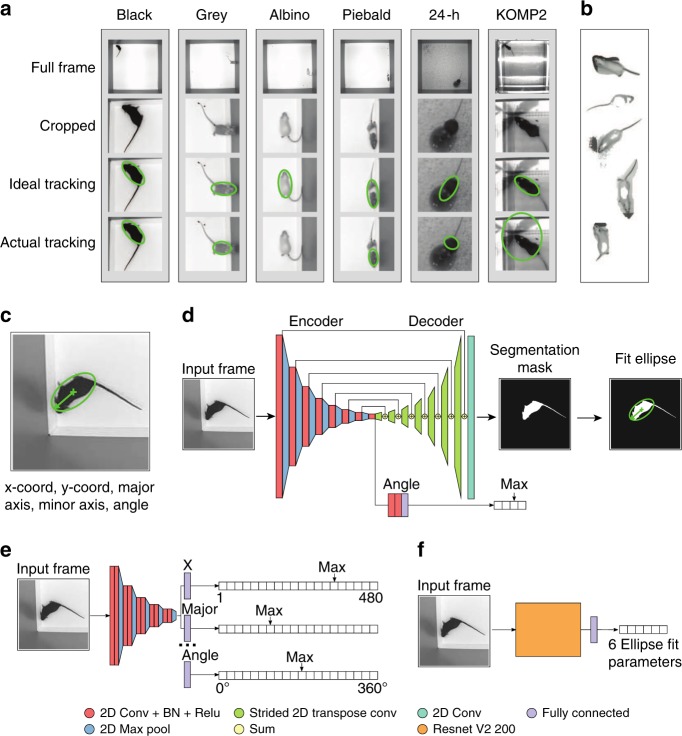
**a** A representation of the environments analyzed by our approaches. A black mouse in a white open field achieves high foreground–background contrast and therefore actual tracking closely matches the ideal. Gray mice are visually similar to the arena walls and therefore often have their nose removed while rearing on walls. Albino mice are similar to the background of the arena itself and are frequently not found during tracking. Piebald mice are broken in half due to their patterned coat color. Placing a food cup into the arena causes tracking issues when the mouse climbs on top. Arenas with reflective surfaces also produce errors with tracking algorithms. **b** We identify the reason for bad tracking to be poor segmentation. Testing a variety of difficult frames with multiple algorithms from the background subtraction library, we do not resolve this segmentation issue. **c** Our objective tracking takes the form of an ellipse description of a mouse. For clarity, we show cropped frames as input into the networks while the actual input is an unmarked full frame. **d** The structure of the segmentation network architecture functions similar to classical tracking approaches in which the network predicts the segmentation mask for the mouse and then fits an ellipse to the predicted mask. **e** The structure of the binned classification network architecture predicts a heatmap of the most probable value for each ellipse-fit parameter. **f** The structure of the regression network architecture directly predicts the six parameters to describe an ellipse for tracking

Our strain survey experiment includes videos of mice with different genetic backgrounds causing expression of different coat colors, including black, agouti, albino, gray, brown, nude, and piebald (Fig. 1a, columns 1, 2, 3, and 4). We tracked all animals in the same white-background open field apparatus. This yielded good results for darker mice (black and agouti mice), but poor results for lighter-colored (albino and gray mice) or piebald mice (Fig. 1a, columns 1, 2, 3, and 4, Supplementary Movie 1). Examples of ideal and actual tracking frames are shown for the various coat colors (Fig. 1a, row 3 and 4 respectively).

We also carried out video analysis of behavior in challenging environments including both 24-h experimental videos that augmented our open field arena, and videos from the open field experiment carried out as part of The Jackson Laboratory KOMP2 (Knockout Mouse Phenotyping Project)^36^ Phenotyping Center (Fig. 1a, column 5 and 6, respectively). In the 24-h experiment, we collected data over multiple days in which mice were housed in the open field with white paper bedding and food cup. The mice were kept in the open field in this multiday data collection paradigm, and continuous recording was carried out in light and dark conditions using an infrared light source. The bedding and food cups were moved by the mouse and the imaging light source alternated between infrared and visible light over the course of each day. The KOMP2 experiment uses a grid of infrared beams to detect a mouses current location (https://www.mousephenotype.org/impress/protocol/81/7). Since the floor of the arena is clear acrylic, the surface of the table on which the arenas were placed shows through as dark gray. In addition, one arena was placed on the junction between two tables, leaving the joint visible. Further, the LED lights overhead caused a very high glare unique to each arena (Supplementary Movie 2). This KOMP2 program has collected over 5 years of data using this system, and we wanted to carry out video-based recording as an added analysis modality to detect gait affects that cannot be identified by beam-break systems. Since environmental alterations could affect the behavioral output and legacy data interpretation, we could not alter the environment for optimal video data collection. Instead, we added a camera on top of each arena. Traditionally, contrast and reflection hurdles could be overcome by changing the environment such that video data collection is optimized for analysis. For instance, to track albino mice, one can increase contrast by changing the background color of the open field to black. However, the color of the environment can effect the behavior of both mice and humans, and such manipulations can potentially confound the experimental results^3,4^. Regardless, such solutions will not work for piebald mice in a standard open field, or any mice in either the 24-h data collection experiment or the KOMP2 arena.

### Identifying source of tracking issues

We found that the combination of mouse coat colors and environments were difficult to handle with Ctrax (Supplementary Movie 1) and LimeLight (Supplementary Movie 3). We carefully optimized and fine-tuned Ctrax for each video (Methods) in each of the three experiments and still found a substantial number frames with poor tracking performance (Fig. 1a, row 4). The frequency of poor tracking instances in an individual video increased as the environment became less ideal for tracking. Furthermore, the distribution of the errors was not random; for example, tracking was highly inaccurate when mice were in the corners, near walls, or on food cups (Fig. 1a, row 4), and less inaccurate when animals were in the center (Supplementary Movie 1). While it is feasible to discard poorly tracked frames, this can lead to biased sampling and skewed biological interpretation.

We explored the cause of bad tracking across our experiments and discovered that, in most cases, improper tracking was due to poor segmentation of the mouse from the background. This included both types of errors: Type I, instances when portions of the background are included as the foreground (e.g. shadows), and Type II, instances when portions of the mouse are removed from the foreground (e.g. albino mouse matching the background color). Since Ctrax uses a single background model algorithm, we tested whether other background model algorithms could improve tracking results. We tested 26 different segmentation algorithms^37^ and discovered that each of these traditional algorithms performs well under certain circumstances but still fail (Fig. 1b), suggesting that there is not a general purpose solution^38^. All currently available mouse tracking software relies on background subtraction approaches (Supplementary Table 1). Since all 26 background subtraction methods from the BGSLibrary failed in some circumstances, we postulate that our results for Ctrax and LimeLight will hold true for these other technologies. In sum, although many video tracking solutions exist, none address the fundamental problem of mouse segmentation appropriately and generally rely on environmental optimization to achieve proper segmentation, therefore creating potential confounds with respect to robust data sampling and analysis. Thus, we could not overcome the fundamental issue of proper mouse segmentation in order to achieve high-fidelity mouse tracking with existing solutions.

A drawback in addition to the problem of inadequate mouse segmentation was the time cost for fine-tuning Ctrax’s settings or another background subtraction algorithm’s parameters. Adjusting the tracking settings for each video added substantial time to our workflow when analyzing thousands of videos. For example, immobile sleeping mice in our 24-h experiment cannot be tracked because the background model incorporates the mouse. Typical supervision (Methods) would take an experienced user 5 min of interaction for each hour of video to ensure high-quality tracking results. While this level of user interaction is tractable for smaller and more restricted experiments, large-scale and long-term experiments are not tractable.

### Proposed neural network solutions

We tested three primary neural network architectures for solving this visual tracking problem (Fig. 1d, e). Each approach attempted to describe the location of the animal through six variables: *x* and *y* location of the mouse in the matrix, major and minor axes of the mouse, and the angle the head is facing (Fig. 1c). To avoid the discontinuity of coterminal angles, the networks predict the sine and cosine of the angle.

The first architecture is an encoder–decoder segmentation network that predicts a foreground–background segmented image from a given input frame (Fig. 1d, Methods). This network predicts which pixels belong to a mouse, with the output being a segmentation mask. After the network produces this segmented image, we applied an ellipse-fitting algorithm for tracking (Supplementary Note 1). Angle direction is selected through an additional cardinal direction output from the network.

The second network architecture is a binned classification network, whereby a probability distribution across a pre-defined range of possible values is predicted for each of the six ellipse-fit parameters (Fig. 1e, Methods). At a resolution of 1 pixel for the *x*-coordinate location of the mouse, there are 480 possible *x*-values (bins) to select from for a 480 × 480 px image. When the network is run, the largest value is selected as the most probable value of the corresponding parameter.

The third architecture is a regression network that predicts the numerical ellipse values directly from the input image (Fig. 1f, Methods). We tested a variety of currently available general purpose feature encoders, and present data from the feature encoder Resnet V2 with 200 convolutional layers, which achieved the best-performing results for this architecture^39^.

### Neural network training

To test the neural network architectures, we built a training dataset of 16,234 training images and 568 held-out validation images across multiple mouse strains and experimental setups (Supplementary Note 2). We created an OpenCV-based labeling interface for creating our training data (Methods) that allows us to quickly annotate foreground and background, and fit an ellipse (Supplementary Figure 1). This annotating interface can be used to quickly generate annotated training data in order to adapt any network to new experimental conditions through transfer learning.

Our network architectures were built, trained, and tested in Tensorflow v1.0, an open-source software library for designing applications that use neural networks^40^. Training benchmarks presented were conducted on the Nvidia P100 GPU architecture. We tuned the hyperparameters through several training iterations. After the first training of networks, we observed that the networks performed poorly under particular circumstances that had not been included in the annotated data, including mid-jump, odd postures, and urination in the arena. We identified and incorporated these difficult frames into our training dataset to further improve performance. A full description of the network architecture definitions and training parameters are available (Methods and Supplementary Table 2).

### Neural network performance

**Fig. 2:**
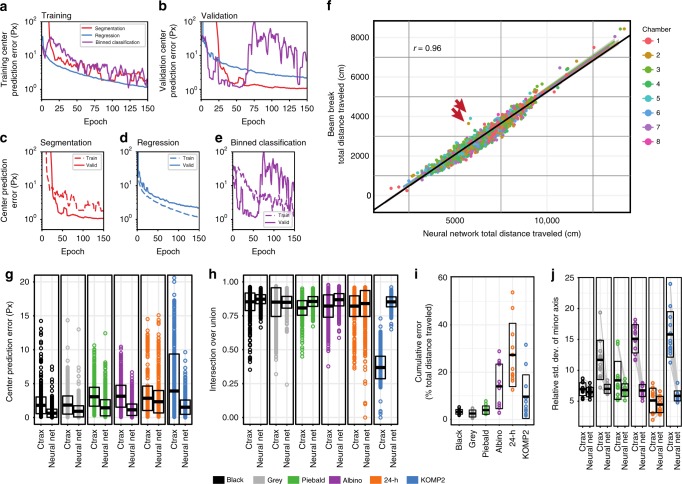
**a**–**e** Performance of our tested network architectures during trainings. **a** Training curves show comparable performance during trainings, independent of network architecture. **b** Validation curves show different performance across the three network architectures. The segmentation network performs the best. **c** Performance increases for validation in our segmentation network architecture. **d** Performance decreases for validation in our regression network architecture, but good generalization performance is maintained. **e** The binned classification network architecture becomes unstable at 55 epochs of training, despite the training curve still improving performance. **f** Comparing our segmentation network architecture with a beam-break system, we observe high Pearson’s correlation. Our network performs consistently, despite the chambers being visually different. We identify two videos that deviate from this trend. **g** Neural network performs better than Ctrax when compared against human annotations. Points indicate annotated frames in a group; bars indicate mean ± standard deviation. **h** Neural network annotations overlap better than Ctrax when compared against human annotations. Ctrax performance on KOMP2 annotated data reveals systematic issue of posture predictions in highly reflective environments. Points indicate annotated frames in a group; bars indicate mean ± standard deviation. **i** Predictions from two approaches yield high agreement on environments with high contrast between the mouse and background (Black, Gray, Piebald). As the segmentation problem becomes more computationally difficult, the relative error increases (Albino, 24 h, KOMP2). Due to low activity in the 24-h setup, errors in tracking have greater influence on the total distance traveled. Points indicate individual videos in a group; bars indicate mean ±standard deviation. **j** Relative standard deviation of the minor axis maintains high correlation when the mouse and environment have high contrast (Black). When segmentation includes shadows, includes reflections, or removes portions of the mouse, the minor axis length is not properly predicted and increases the relative standard deviations (Gray, Piebald, Albino, KOMP2). Points indicate individual videos in a group; bars indicate mean ± standard deviation

Overall, training and validation loss curves indicated that each of the three network architectures trains to a performance with an average center location error between 1 and 2 pixels (Fig. 2a). The encoder–decoder segmentation architecture converged to a validation center location error of 0.9 px (Fig. 2a–c). Surprisingly, we found the binned classification network displayed unstable loss curves, indicating overfitting and poor generalization (Fig. 2b, e). The regression architecture converged to a validation center location error of 1.2 px, showing a better training than validation performance (Fig. 2a, b, d). We further investigated the performance of posture predictions on the segmentation and regression networks. A commonly used metric for this task is calculating the Intersection over Union (IoU) score for a prediction relative to a ground truth to identify the overlap. The encoder–decoder segmentation network achieved a mean IoU score of 0.828 on validation while the regression network achieved a mean IoU score of 0.743. While both scores are considered acceptable, the encoder–decoder segmentation network has a clear improvement on posture abstraction.

The encoder–decoder segmentation architecture performs well in both accuracy and computational efficiency, requiring an average processing time of 5–6 ms per frame. Our video data could be processed by this network at a rate of up to 200 frames per second (fps) (6.7× real time) on a Nvidia P100, which is a server-grade GPU.; and a rate of up to 125 fps (4.2× real time) on a Nvidia TitanXP, a consumer-grade GPU. In comparison, Resnet V2 200 incurs a substantially longer processing time per frame (33.6 ms on a Nvidia P100). Other pre-built general-purpose networks^41^ achieve similar or worse performances at a tradeoff of faster compute time. Thus, regression networks are an accurate but computationally expensive solution for long-term video analysis.

### Training set comparisons

We also tested the minimum training dataset size required to train the encoder–decoder segmentation network, by randomly subsetting our training dataset to smaller numbers of annotated images (10,000 to 500) and training the network from scratch. We obtained good results from a network trained with only 2500 annotated images, a task that takes approximately 3 h to generate with our labeling interface (Supplementary Figure 2).

Additionally, we tested the influence of inclusion for different training data subsets. While a general network can be trained with all data, slightly improved performance is obtained through training models specific to the experimental environment (Supplementary Tables 3 and 4).

### Neural network comparison with traditional systems

We evaluated the quality of the encoder–decoder segmentation neural network tracking architecture by inferring entire videos from mice with disparate coat colors and data collection environments (Fig. 1a) and visually evaluating the quality of the tracking. We also compared this neural network-based tracking architecture with an independent modality of tracking, the KOMP2 beam-break system (Fig. 1a, column 6). We tracked 2002 videos of individual mice comprising 700 h of video from the KOMP2 experiment using the encoder–decoder segmentation neural network architecture and compared the results with the tracking data obtained using the KOMP2 beam-break system (Fig. 2f). These data comprised mice of 232 knockout lines on the C57BL/6NJ background that were tested in 20-min open field assay in 2016 and 2017. Since each KOMP2 arena has slightly different background due to the transparent and reflective walls, we compared tracking performances of the two approaches for each of the eight testing arenas used in the 2016 and 2017 KOMP2 open-field assays (Fig. 2f, colors shows arena), and compared tracking performances for all the arenas combined (Fig. 2f, black line). We observed a very high correlation between the total distance traveled in the open field as measured by the two approaches across all eight KOMP2 testing arenas (*R* = 96.9%, Fig. 2f). We observed two animals with high discordance from this trend (Fig. 2f, red arrows). Observation of the video showed odd behaviors for both animals, with a waddle gait in one and a hunched posture in the other (Supplementary Movie 2). We postulate that these behaviors led to an erroneously high number of beam breaks in the beam-break system. This example highlights an important advantage of the neural network, as it is unaffected by the behavior of the animal.

We then compared the performance of our trained segmentation neural network with the performance of Ctrax across a broad selection of videos from the various testing environments and coat colors previously tracked using Ctrax and LimeLight (Fig. 1a). We wish to emphasize that we compared the performance of our network with that of Ctrax because Ctrax is one of the best conventional tracking software packages that allows fine tuning of the many tracking settings, is open source, and provides user support. Given the results with the 26 background subtraction approaches (Fig. 1b), we expected similar or worse performances from other tracking systems. We hand annotated 7200 ground truth test frames across six videos with one animal per group (Fig. 1a). Each ground truth was annotated at 1 s intervals, achieving a 20-min span of activity per video. We compared the predictions generated from Ctrax and our trained encoder–decoder segmentation neural network. Videos in which the mouse was immobile for a long duration and incorporated into Ctrax’s background model were manually corrected. We calculated the center hypotenuse error and found that our neural network produced significantly better prediction accuracies across increasingly difficult environments (Fig. 2g, Supplementary Note 3, Supplementary Table 5). Even though the neural network has never been trained with images of annotated piebald coat color mice, the network has generalized to track these mice with better accuracy than Ctrax. We observed improved IoU performance using the neural network over Ctrax for all environmental groups except gray mice, where performance only reduced the standard deviation (Fig. 2h). Surprisingly, in the KOMP2 setup, Ctrax’s performance substantially dropped compared to the other environments. This performance drop is due to the high occurrence of reflections and non-uniform lighting, factors known to be difficult for Ctrax’s algorithm. While the *x*−*y* error seems reasonable for Ctrax in the KOMP2 setup, Ctrax is unable to adequately abstract the posture of the mouse to an accurate ellipse, as shown by a mean IoU of 0.37.

While these errors may appear insignificant for tracking, we analyzed more videos at higher temporal resolution to further investigate how these differences impact behavioral metrics. We tracked 72 videos, broken into six groups (Fig. 1a) with 12 animals per group, with both our trained encoder–decoder segmentation neural network and Ctrax. We calculated a cumulative relative error of total distance traveled between Ctrax and our neural network (Fig. 2i). Specifically, for every minute in the video, we compared the distance-traveled prediction of the neural network with that of Ctrax. Tracking for black, gray, and piebald mice in the white-background open-field apparatus showed errors less than 4%; however, higher levels of error were seen in albino mice in the open-field arena with a white floor (14%), black mice in the 24-h arena (27%), and black mice in the KOMP2 testing arena (10%) (Fig. 2i, Supplementary Movie 1). Despite only a minor difference in center location error for albino mice (Fig. 2g, Ctrax center error mean of 3.1 px and Neural Network center error mean of 1.1 px), the two methods disagree greatly for behavioral metrics (Fig. 2i, 4 of 12 videos greater than 20% disagreement). Thus, we could not adequately track albino mice in the open-field arena with a white floor, black mice in the 24-h arena, or black mice in the KOMP2 testing arena without the neural network tracker.

We also observed, using Ctrax, that when foreground segmentation prediction is incorrect, such as when shadows are included in the prediction, the ellipse fit does not correctly represent the posture of the mouse (Supplementary Movie 1). In these cases, even though the center of mass tracking was acceptable, the ellipse fit itself was highly variable. Modern machine learning software for behavior recognition, such as the Janelia Automatic Animal Behavior Annotator (JAABA)^42^, utilize the time series of ellipse-fit tracking values for classification of behaviors. When tracking is accurate, the relative standard deviation of the minor axis shows the least variance across all sizes of laboratory mice, as the width of an individual mouse remains similar through a wide range of postures expressed in behavioral assays. We observed a higher variation with Ctrax for gray and piebald mice in the white open field arena (Fig. 2j) even though there is low cumulative relative error of total distance traveled (Fig. 2i). This increased variance in minor axis observed in Ctrax is due to instability of tracking caused by poor segmentation, as suggested by IoU on ground truth labels (Fig. 2h). This inaccurate posture estimation adversely affects automated behavior system predictions.

### Large-scale experiments

**Fig. 3:**
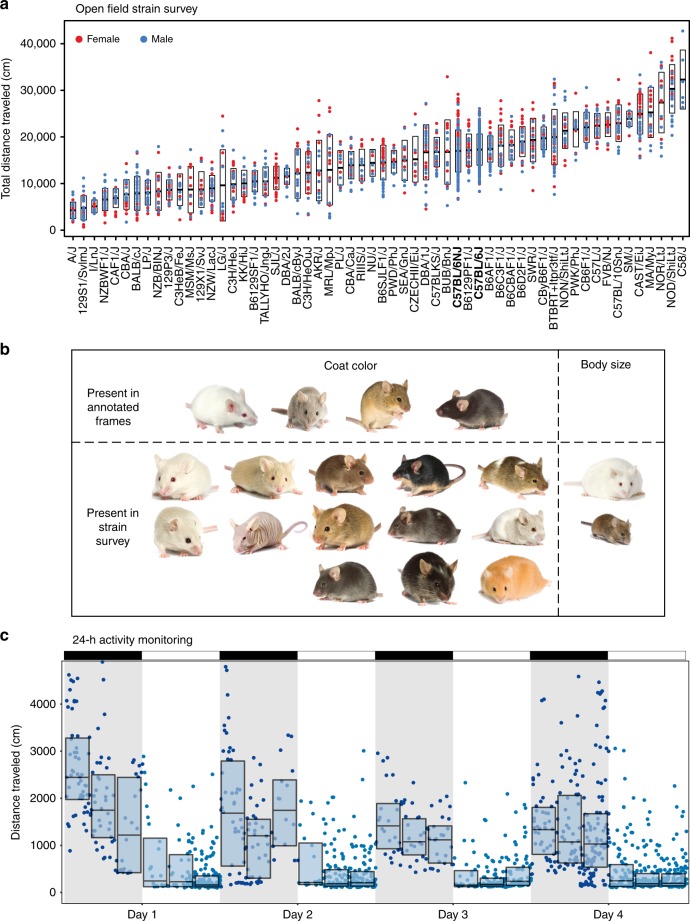
Highly scalable tracking with a single neural network. **a** A large strain survey showing genetically diverse animals tracked with our encoder–decoder segmentation network. In total, 1845 animals across 58 inbred and F1 isogenic strains, totaling 1691 h of video, were processed by a single trained neural network. Total distance traveled in a 55-min open field assay is shown. Points indicate individuals in a strain; box indicates mean ±standard deviation. Two reference mouse strains are shown in bold, C57BL/6J and C57BL/6NJ. **b** Representation of visual variation for track-able mice in the strain survey. Our network was trained on a small subset of actual variation in visual appearance (Image credit: JAX Creative). **c** Daily activity rhythms observed in six animals continuously tracked over 4 days in a dynamic environment with our encoder–decoder neural network. Points indicate distance traveled in 10 min intervals. Boxes indicate quantiles for 4-h intervals. Light bar and gray background indicate light–dark cycle

Having established the encoder–decoder segmentation neural network as a highly accurate tracker, we tested its performance using two large behavioral experiments. For the first experiment, we generated white-surfaced open-field video data with 1845 mice, including 58 strains of mice including mice with diverse coat colors, piebald mice, nude mice, and obese mice, and covering a total of 1691 h (Fig. 3a). This dataset consists of 47 inbred strains and 11 isogenic F1 strains and is the largest open-field dataset generated, based on the data in the Mouse Phenome Database^43^. Using a single trained network, we were able to track all mice with high accuracy. Our network achieved strong generalization, being able to track a broad spectrum of mouse coat colors and body shapes without requiring additional frame annotations (Fig. 3b). We visually checked mice from a majority of the strains for fidelity of tracking and observed excellent performance. These results are compared with three other open-field activity phenotype datasets, including Tarantino (MPD 50601), Pletcher (MPD 36007), and Wiltshire (MPD 21401). The Tarantino dataset^44^ includes 37 strains in a 10-min open field with only females. The Pletcher dataset^45^ includes 31 strains in a 10-min open field with only males. The Wiltshire dataset includes 37 strains in a 10-min open field with both sexes. The activity phenotypes that we observed are concordant with previously published datasets of mouse open-field behavior (Supplementary Figure 3).

For the second dataset, we tracked 24-h video data collected for four C57BL/6J and two BTBR T^+^ ltpr3^tf^/J mice (Fig. 1a, column 5). These mice were housed with bedding and a food cup over multiple days during which the food changed location and under 12:12 light–dark conditions (Methods). We tracked activity across all animals under these conditions using our neural network and observed very good performance under both light and dark conditions (Fig. 3c). As expected, we observed daily activity rhythm with high levels of locomotor activity during the dark phase.

### Environmental condition testing

In addition to these two large-scale experiments, we also conducted two experiments to showcase the robustness of our system over existing tracking solutions. The first experiment observes an albino mouse in a white arena with changing lighting conditions (Supplementary Movie 4). Single background model-based tracking solutions are insufficient for adequately tracking in this experiment (Supplementary Figure 4a), only being able to detect a mouse 46.3% of the time (Supplementary Figure 4a, left bar plot). Even when this detection rate is ignored, about 10% of the comparible frames expose shadow-related issues for Ctrax in this suboptimal environment (Supplementary Figure 4a, right histogram). Our neural network solution, due to augmentation during training, requires no newly annotated frames to successfully track in this experiment (Supplementary Figure 4a, example frames).

The second experiment adds large moveable objects to the arena for the mouse to investigate and interact (Supplementary Movie 5). This simulates a dynamic environment where non-mouse objects are also moving. A single background model tracking solutions is insufficient for tracking this experiment (Supplementary Figure 4b), often confusing the moving objects as other mice (Supplementary Figure 4b, example frames). In this video, where the mouse happens to seldomly interact with the objects, about 10% of the frames contain tracking swaps between sphere and the mouse (Supplementary Figure 4b, right histogram). A neural network, trained with only 500 annotations from separate videos, is still able to generalize well for tracking mice interacting with these spheres in arbitrary configurations (Supplementary Movie 5).

## Discussion

Video-based tracking of animals in complex environments has been a long-standing challenge in the field of animal behavior^1^. Current animal-tracking systems do not address the fundamental issue of animal segmentation and rely heavily on visual contrast between the foreground and background for accurate tracking. As a result, the experimenter must restrict the environment to achieve optimal results. Here we describe a modern neural network-based tracker that addresses a fundamental issue in laboratory animal tracking—environment restrictions for foreground and background segmentation. We test three different architectures and find that an encoder–decoder segmentation network architecture achieves the highest level of accuracy and functions at a high speed (over 6× real time). Furthermore, we provide an annotating interface that allows the user to train a new network for their specific environment by annotating as few as 2500 images, which takes approximately 3 h. We compare our network to two existing solutions and find that it vastly outperforms them in complex environments and expect similar results with systems that utilize background subtraction approaches. In fact, when we tested 26 different background subtraction methods we discovered that each failed under certain circumstances. However, a single neural network architecture functions for all coat colors of mice under multiple environments. We also show that our neural network approach is robust to dynamic experimental setups that would cause traditional approaches to fail. Our machine learning approach enables long-term tracking under dynamic environmental conditions with minimal user input, thus establishing the basis of the next generation of tracking architecture for behavioral research.

Our approach also exhibits good generalization capabilities. A trained model can generalize to work on new environments, perspectives, and mice as long as the task is visually similar. This is apparent by observing good tracking performance on piebald mice without having any annotations shown during training (Supplementary Table 5) and the dynamic lighting experiment (Supplementary Figure 4a). However, to ensure good performance on data, we recommend testing sample data and annotating new frames accordingly. While we observe good performance with 2500 annotated frames, transfer learning from a model pretrained on our annotated dataset can reduce this cost.

Our proposed solution specifically addresses needs that have been identified by machine learning experts in the field of animal tracking^5^—development of technologies that are simple to use, can track animals without markings, function with diverse range of animal visual appearance, function in diverse natural environments, minimizes data handling, utilizes a single image point (single camera), and can automate behavior detection. The work presented here is able to track a wide variety of mice with varying coat colors, body shapes, and sizes and thus can function with a very diverse range of animal appearance. We are also able to track single mice in a wide variety of environmental conditons over long periods of time with minimal supervision using a single video perspective. Furthermore our methods can be integrated into other systems to address simplicity, data handling, and behavior detection. The ability to track diverse range of mice under diverse environmental condiditons are critical for ethologically relevant assays inside and outside the laboratory. These improvements enable complex behavioral experimentation with advanced mouse populations such as Diversity Outcross (DO) and Collaborative Cross mice^26^. DO mice are designed to model genetic diversity seen in human populations and characteristically have highly diverse visual appearance (body size, coat color) and behavioral repertoires. Thus, we propose a solution that address key deficits in current animal-tracking technology and will enable large-scale behavioral analysis under varied environmental conditions.

## Methods

### Experimental arenas

*Open field arena*: Our open field arena measures 52 cm by 52 cm by 23 cm. The floor is white PVC plastic and the walls are gray PVC plastic. To aid in cleaning maintenance, a white 2.54 cm chamfer was added to all the inner edges. Illumination is provided by an LED ring light (Model: F&V R300). The ring light was calibrated to produce 600 lux of light in each of our 24 arenas.

*24-h monitoring open field arena*: We augmented six of our open field arenas for multiple day testing. We set our overhead LED lighting to a standard 12:12 light–dark cycle. ALPHA-dri was placed into the arena for bedding. To provide food and water, a single Diet Gel 76A food cup was placed in the arena. This nutritional source was monitored and replaced when depleted. Each arena was illuminated at 250 lux during the day and <5 lux during the night. For recording videos during the night, additional IR LED (940 nm) lighting was added.

*KOMP2 open field arena:* In addition to our custom arenas, we also benchmarked our approach on a commercially available system. The Accuscan Versamax Activity Monitoring Cages is constructed using clear plastic walls. As such, visual tracking becomes very difficult due to the consequent reflections. The cage measures 42 cm by 42 cm by 31 cm. Lighting for this arena was via LED illumination at 100–200 lux.

We use the beam-break system as a commercial turn-key system that is well established in the specific field for tracking rodents in empty environments. The tracking system used by KOMP2 at JAX is sold by Omnitech Electronics Inc. (http://omnitech-usa.com/product/Open-Field---Locomotor-Activity/1010) and is described in detail a the International Mouse Phenotyping Center (https://www.mousephenotype.org/impress/protocol/81/7). Briefly, this system creates a grid of IR beams that are approximately 1 inch apart in the *x*, *y* direction and a second set of beams on the *z*-axis to detect rearing. The location and speed of mouse movement is tracked by the grid location of interrupted beams.

### Lighting test in open field arena

For the lighting test, we used our open field arena. We disabled the camera target brightness during recording and manually adjusted the LED illumination lamps to a variety of lighting conditions between 300 lux and 1000 lux. This corresponds to greyscale range of values of 140–250 for the center color (normally white-balanced to 225) and a wall greyscale range of 75–150 (normally white-balanced to 132). A single NOD/ShiLtJ mouse was placed in the arena and recorded for 10 min.

### Sphere test in open field arena

For the move-able sphere test, we used our open field arena and added reflective spheres of varying sizes into the arena with the mouse. The spheres are 20 swg 304 stainless steel hollow spheres measuring 6, 8, and 10 cm diameter. These spheres are light enough for a mouse to move them around with ease. Two to five spheres were placed into the arena at random starting locations for recording. We recorded six videos, one of which was held out for validation. Each video was 40 min. One DBA/2J mouse was placed in each arena for recording.

### Video acquisition

All data were acquired using the same imaging equipment. Data were acquired at 640 × 480 px resolution, 8-bit monochrome depth, and 30 fps using Sentech cameras (Model: STC-MB33USB) and Computar lenses (Model: T3Z2910CS-IR). Exposure time and gain were controlled digitally using a target brightness of 190/255. Aperture was adjusted to its widest so that lower analog gains were used to achieve the target brightness. This in turn reduced amplification of baseline noise. Files were saved temporarily on a local hard drive using the “raw video” codec and “pal8” pixel format. Our typical assays run for 2h, yielding a raw video file of approximately 50 GB. Overnight, we use FFmpeg software (https://www.ffmpeg.org/) to apply a 480 × 480 px crop, de-noise filter, and compress using the mpeg4 codec (quality set to max) using the YUV420P pixel format, which yields a compressed video size of approximately 600 MB.

One camera and lens was mounted approximately 100 cm above each arena to alleviate perspective distortion. Zoom and focus were set manually to achieve a zoom of 8 px/cm. This resolution both minimizes the unused pixels on our arena border and yields approximately 800 pixels area per mouse. Although the KOMP2 arena is slightly smaller, the same zoom of 8px/cm target was utilized.

### Ctrax supervision protocol

Ctrax contains a variety of settings to enable optimization of tracking^28^. The authors of this software strongly recommend, first and formost, ensuring that the arena is set up under specific criteria to ensure good tracking. In most of our tests, we intentionally use an environment in which Ctrax is not designed to perform well (e.g., albino mice on a white background). That being said, with well-tuned parameters, a good performance is still achievable. However, with a large number of settings to manipulate, Ctrax can easily require substantial time to achieve a good tracking performance. Here, we describe our protocol for setting up Ctrax for tracking mice in our environments.

First, we create a background model. The core of Ctrax is based on background subtraction, and thus a robust background model is essential for functionality. Models function optimally when the mouse is moving. To create the background model, we seek to a segment of the video in which the mouse is clearly moving, and we sample frames from that section. This ensures that the mouse is not included in the background model. This approach improves Ctrax’s tracking performance on our 24-h data, as the mouse moves infrequently due to sleeping and would typically be incorporated into the background model.

The second step is to set the settings for background subtraction. Here, we use the background brightness normalization method with a std range of 254.9–255.0. The thresholds applied to segment out the mouse are tuned on a per-video basis, as slight changes in exposure and coat color will influence the performance. To fine-tune these thresholds, we apply starting values based on previous videos analyzed and adjust values by checking multiple portions of the video. Every video is inspected for proper segmentation on difficult frames, such as the mouse rearing on the wall. Additionally, we apply morphological filtering to both remove minor noise in the environment as well as remove the tails of mice for fitting an ellipse. We use an opening radius of 4 and a closing radius of 5.

Lastly, we manually set a variety of tracking parameters that Ctrax enables to ensure that the observations are in fact mice. For optimal time efficiency, these parameters were tuned well once and then used for all other mice tracked. If a video was performing noticeably poorly, the general settings were tweaked to improve performance. For the shape parameters, we computed bounds based on two standard deviations from an individual black mouse video. We lowered the minimum values further because we expected that certain mice would perform poorly on the segmentation step. This allows Ctrax to still find a good location of the mouse despite not being able to segment the entire mouse. This approach functions well, as all of our setups have the same zoom of 8, and the mice tested are generally the same shape. Motion settings are very lenient, because our experimental setup tracks only one mouse in the arena at a time. Under the observation parameters, we primarily utilize the “Min Area Ignore” setting to filter out detections larger than 2500 pixels. Under the hindsight tab, we use the “Fix Spurious Detections” setting to remove detections with a length shorter than 500 frames.

### Training set annotation

We annotated our own training data using custom software that was written to accommodate obtaining the necessary labels. We used the OpenCV library (https://opencv.org/) to create an interactive watershed-based segmentation and contour-based ellipse-fit. Using the software GUI we developed, the user left-clicks to mark points as the foreground (a mouse) and right-clicks to label other points as the background (Supplementary Figure 1). Upon a keystroke, the watershed algorithm is executed to predict a segmentation and ellipse. If users need to make edits to the predicted segmentation and ellipse, they can simply mark additional areas and run the watershed again. When the predictions are of sufficiently high quality, users then select the direction of the ellipse. They do this by selecting one of four cardinal directions: up, down, left, and right. Since the exact angle is selected by the ellipse-fitting algorithm, users need only to identify the direction ±90°. Once a direction is selected, all the relevant data are saved to disk and users are presented with a new frame to label. Full details on the software controls can be found in the software documentation.

The objective of our annotated dataset is to identify good ellipse-fit tracking data for mice. While labeling data, we optimized the ellipse-fit such that the ellipse was centered on the mouse’s torso with the major axis edge approximately touching the nose of the mouse. Frequently, the tail was removed from the segmentation mask to provide a better ellipse-fit. For training networks for inference, we created three annotated training sets. Each training dataset includes a reference frame (input), segmentation mask, and ellipse-fit. Each training set was generated to track mice in a different environmental setup.

### Neural network model description

The neural networks we trained fall into three categories: segmentation, regression, and binning. Our tested models can be viewed visually in Fig. 1d–f.

The first network architecture is modeled after a typical encoder–decoder structure for segmentation (Fig. 1d). The primary structure of this architecture starts with a feature encoder, which abstracts the input image down into a small-spatial-resolution set of features. The encdoded features are then passed to both a feature decoder, which converts this set of features back into the same shape as the original input image, and three fully connected layers to predict, which cardinal direction the ellipse is facing. We trained the feature decoder to produce a foreground–background segmented image. The first half of the network (encoder) utilizes 2D convolutional layers followed by batch normalization, a ReLu activation, and 2D max pooling layers. We use a starting filter size of 8 that doubles after every pooling layer. The kernels used are of shape 5 × 5 for 2D convolution layers and 2 × 2 for max pooling layers. Our input is of shape 480 × 480 × 1 and after six of these repeated layers, the resulting shape is 15 × 15 × 128. We apply another 2D convolutional layer (kernel 5 × 5, 2× filters) followed by a 2D max pool with a different kernel of 3 × 3 and stride of 3. One final 2D convolutional layer is applied to yield our feature bottleneck with a shape of 5 × 5 × 512. This feature bottleneck is then passed to both the segmentation decoder and angle predictor. The segmentation decoder reverses the encoder using strided transpose 2D convolutional layers and carries over pre-downsampled activations through summation junctions. It should be noted that this decoder does not utilize ReLu activations. After the layers return to the 480 × 480 × 8 shape, we apply one additional convolution, with a kernel size of 1 × 1, to merge the depth into two images: background prediction and foreground prediction. We apply a softmax function across this depth. From the feature bottleneck, we also create a prediction for angle prediction. We achieve this by applying two 2D convolution layers with batch normalization and ReLu activations (kernel size 5 × 5, feature depths 128 and 64). From here, we flatten and use one fully connected layer to yield a shape of four neurons, which function to predict the quadrant in which the mouse’s head is facing. Since the angle is predicted by the mask, we need only to select the correct direction (±180°). The four possible directions that the network can select are 45–135, 135–225, 225–315, and 315–45° on a polar coordinate grid. These boundaries were selected to avoid discontinuities in angle prediction. Two losses are used, a softmax cross entropy for segmentation mask prediction and a second softmax cross entropy for cardinal angle prediction. During training, these losses are summed.

The second network architecture is a binned regression approach (Fig. 1e). Instead of predicting the parameters directly, the network instead selects the most probable value from a selection of binned possible values. The major difference between this structure and a regression structure is that the binned regression network training relies on a cross entropy loss function whereas a regression network relies on a mean squared error loss function. Due to memory limitations, we tested only custom VGG-like networks with reduced feature dimensions. This network architecture begins with a feature encoder that abstracts the input image down into a small-spatial-resolution set of features. The encoded features are flattened and connected to additional fully connected layers whose output shape is determined by the desired resolution of the output. Our best-performing network is structured with two 2D convolutional layers followed by a 2D max pooling layer. The kernels used are of shape 3 × 3 for 2D convolutional layers and 2 × 2 for 2D max pooling layers. We start with a filter depth of 16 and double after every 2D max pool layer. This two convolutional plus max pool sequence is repeated five times to yield a shape of 15 × 15 × 256. This layer is flattened and connected to a fully connected layer for each output ellipse-fit parameter. The shape of each output is dictated by the desired resolution and range of the prediction. Each desired output parameter is realized as an independent set of trainable fully connected layers connected to the encoded features. For testing purposes, we observed only the center location and trained with a range of the entire image (0–480). Additional outputs, such as angle prediction, could simply be added as additional output vectors. Training this network uses a categorical cross entropy loss for each parameter prediction. During training, all parameter losses are summed.

The third network architecture is modeled after a typical regression predictor structure (Fig. 1f). While the majority of regression predictors realize the solution through a bounding box, an ellipse simply adds one additional parameter: the angle of the mouse’s head direction. Since multiple angles can be coternimal with discontinuous equivalence at 360° and 0°, we transform the angle parameter into its sine and cosine components. This yields a total of six parameters regressed from the network. The network architecture begins with a feature encoder that abstracts the input down into a small spatial resolution. These encoded features are then flattened and connected to fully connected layers to produce an output shape of 6, the number of values that we ask the network to predict to fit an ellipse. We tested a wide variety of pre-built feature detectors including Resnet V2 50, Resnet V2 101, Resnet V2 200, Inception V3, Inception V4, VGG, and Alexnet. In addition to these pre-built feature detectors, we also surveyed a wide array of custom networks. Of these general purpose feature encoders and custom networks, Resnet V2 200 performed the best. Training this network uses a mean squared (L2) loss where the squared difference between prediction and ground truth are calculated.

### Neural network training

This section describes all of the procedures pertaining to training our neural network models. The three procedures described here are training set augmentation, training hyperparameters, and a benchmark for training set size.

Training set augmentation has been an important aspect of training neural networks since Alexnet^46^. We utilize a handful of training set augmentation approaches to achieve good regularization performance. Since our data are from a birds-eye view, it is straightforward to apply horizontal, vertical, and diagonal reflections for an immediate 8× increase in our equivalent training set size. Additionally, we apply small rotations and translations for the entire frame. Rotation and translation augmentation values are sampled from a uniform distribution. Finally, we apply noise, brightness, and contrast augmentations to the frame. The random values used for noise, brightness, and contrast augmentations are sampled from a normal distribution.

Hyperparameters, such as training learn rate and batch size, were selected independently for each network architecture trained. While larger networks, such as Resnet V2 200, can run into memory limitations for batch sizes at an input size of 480 × 480, good learning rate and batch size were experimentally identified using a grid search approach^47^. Supplementary Table 2 summarizes all the hyperparameters selected for training these network architectures.

We also benchmarked the influence of training set size on network generalization in order to determine the approximate amount of annotated training data required for good network performance of the encoder–decoder segmentation network architecture (Supplementary Figure 2). We tested this benchmark by shuffling and randomly sampling a subset of the training set. Each subsampled training set was trained and compared to an identical validation set. While the training curves appear indistinguishable, the validation curves trained with fewer than 2500 training annotations diverge from the group. This suggests that the training set is no longer large enough to allow the network to generalize well. While the exact number of training samples will ultimately rely on the difficulty of the visual problem, a recommended starting point would be around 2500 training annotations.

Finally, we compare network performance on the training and validation datasets based upon which training annotations were included during the training process (Supplementary Tables 3 and 4). We train five separate models, three of which are used in the main text figures. The Full Model utilizes all available annotations across our three datasets (Supplementary Note 2). The No Difficult Frames Model excludes only the “difficult frame” annotations to show performance changes when annotating additional frames found to be incorrect outside the annotated dataset. The remaining models are trained only on the subset of performance metrics available. We observe slightly better performance by only training on a single dataset grouping when compared to a model trained on all annotations. Our annotated test data in Supplementary Table 5 suggest that either approach (training 1 model on all annotations or multiple models grouped by environment) outperforms Ctrax.

### Animals used

All animals were obtained from The Jackson Laboratory production colonies. Adult mice aged 8 to 14 weeks were behaviorally tested in accordance with approved protocols from The Jackson Laboratory Institutional Animal Care and Use Committee guidelines. Open field behavioral assays were carried out as previously described^48^. Briefly, group-housed mice were weighed and allowed to acclimate in the testing room for 30–45 min before the start of video recording. Data from the first 55 min of activity are presented here. Where available, eight males and eight females were tested from each inbred strain and F1 isogenic strain.

### Reporting summary

Further information on experimental design is available in the Nature Research Reporting Summary linked to this article.

## Supplementary information

Supplementary Information

Description of Additional Supplementary Files

Reporting Summary

Supplementary Movie 1

Supplementary Movie 2

Supplementary Movie 3

Supplementary Movie 4

Supplementary Movie 5

## Data Availability

The neural network training code used in this study is available in a github repository https://github.com/KumarLabJax/MouseTracking. The annotation tool code as well as supporting scripts is available in a github repository https://github.com/KumarLabJax/MouseTrackingExtras.

## Data availability

Neural network training sets used in this study are available on the kumarlab website https://www.kumarlab.org/2019/02/12/single-mouse-tracking-annotated-dataset/. Pretrained neural networks used in this study are available on the kumarlab website https://www.kumarlab.org/2019/02/12/pre-trained-single-mouse-tracking-neural-network-models/. Strain survey distance traveled data used in this study are available in MPD.
