# Supplementary material for: Robust mouse tracking in complex environments using neural networks
Source: Commun Biol. 2019 Mar 29;2:124. doi: 10.1038/s42003-019-0362-1 (PMC6440983; doi:10.1038/s42003-019-0362-1)
Supplement: Supplementary file 1 — Supplementary Information [file 42003_2019_362_MOESM1_ESM.pdf]

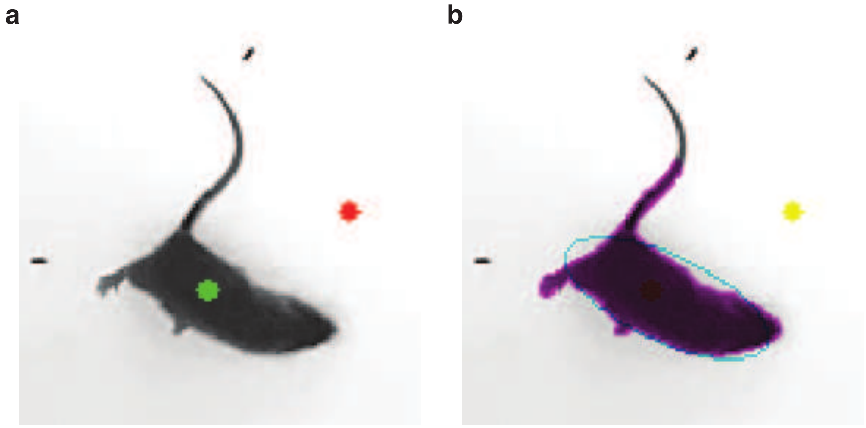

Supplementary Figure 1: An example of our labeling GUI software. (a) shows that the user has zoomed into the mouse and placed 2 marks for foreground (green) and background (red). (b) shows the resulting segmentation (magenta), ellipse-fit (cyan), and old background labels (yellow).

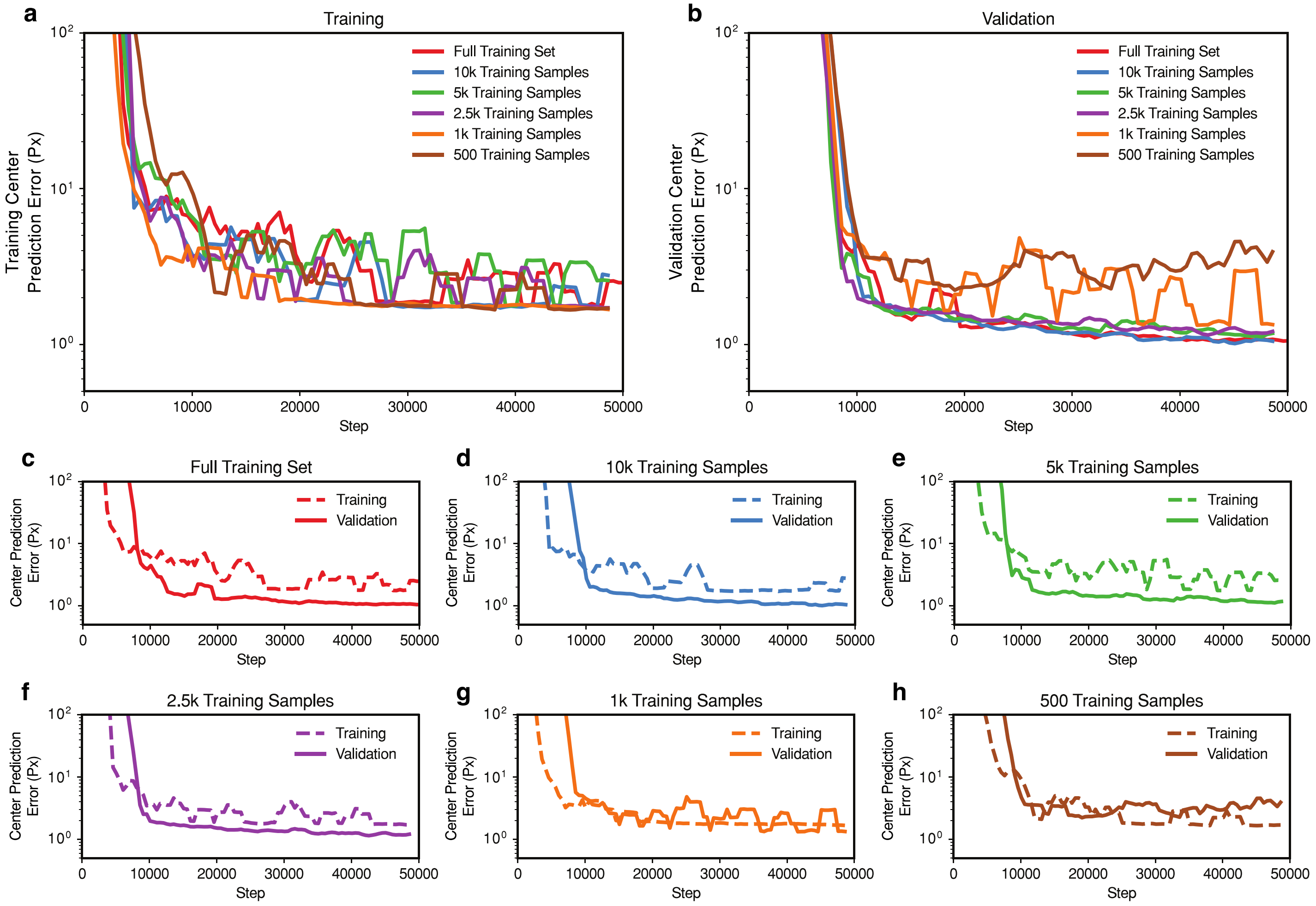

Supplementary Figure 2: We benchmarked how the training set size influences the performance of a trained encoder-decoder segmentation network. (a) Training set size shows no performance change in training set error rate. (b) Validation performance converges to the same value above 2,500 training samples but error is increased below 1,000 training samples. (c-f) Validation accuracy outperforms training accuracy while above 2,500 training samples. (g) Validation accuracy begins to show signs of weak generalization only matching training accuracy at 1,000 training samples. (h) A network trained using only 500 training samples is clearly overtraining, shown by the diverging and increasing validation error rate.

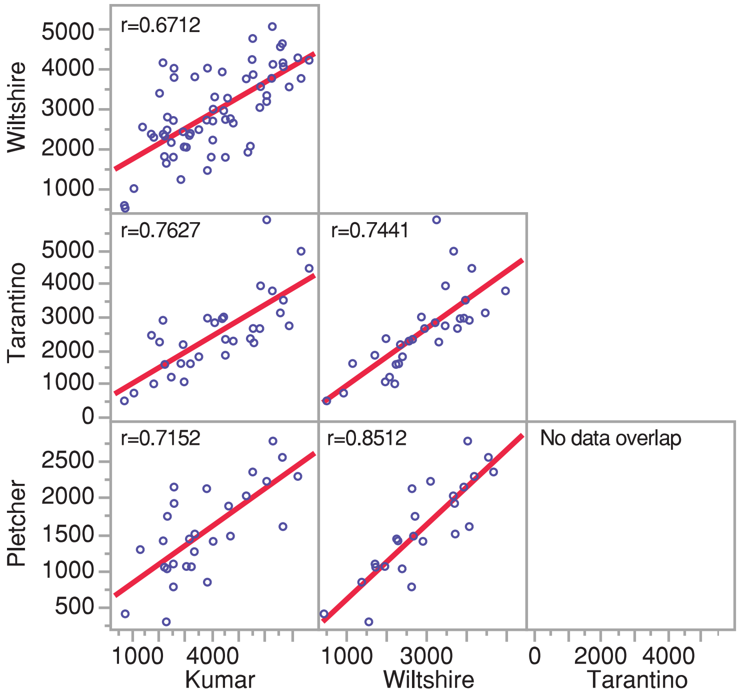

Supplementary Figure 3: We compare our strain survey dataset with multiple external datasets (Pletcher MPD36007, Tarantino MPD50601, and Wiltshire MPD21401). We recalculated our data to analyze total distance traveled in the first 10 minutes of the open field assay in order to be consistend with the three datasets deposited in Mouse Phenome Database (https://phenome.jax.org/). We created correlation scatterplots of these datasets to calculate Pearson's correlation coefficient r=0.67, r=0.72, r=0.76 for Wiltshire, Tarantino, and Pletcher datasets, respectively. This was within the range of correlation coefficients seen in the comparison of the three published datasets among themselves (r=0.74 to r=0.85).

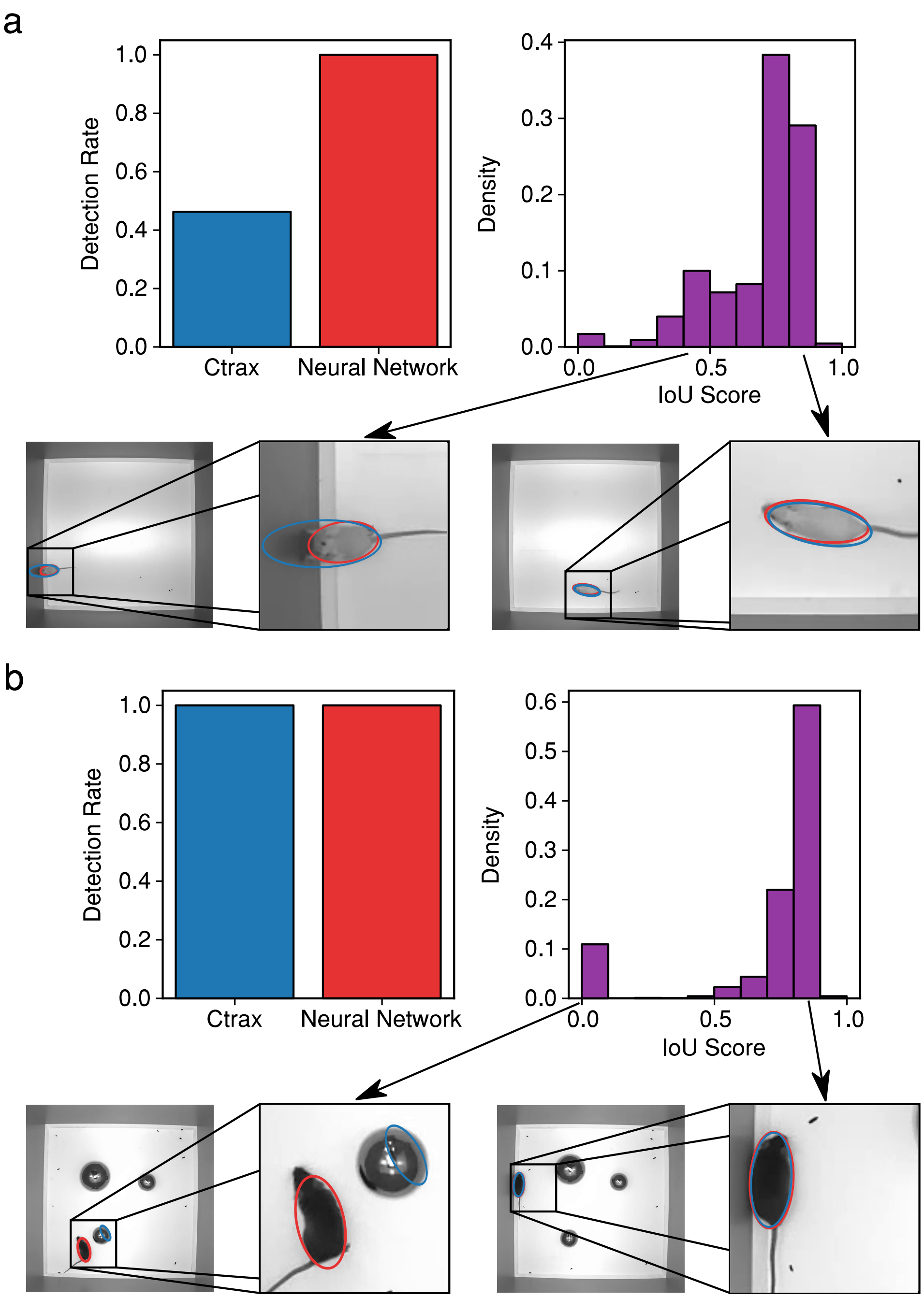

Supplementary Figure 4: Robustness testing of tracking approaches in dynamic conditions. (a) An experiment that involves dynamic lighting poses as an extremely difficult task for tracking algorithms that rely upon background models. Ctrax is only able to detect a mouse in 46% of the frames, while the neural network predicts an ellipse for every frame. Comparing only the frames where both approaches make a prediction shows that Ctrax achieves poor performance on an additional 16% of frames. Example frames show Neural Network predictions (red) and Ctrax predictions (blue). (b) An experiment that involves additional dynamic objects in the arena. Ctrax predicts multiple objects in the arena, but non-mouse tracks were filtered out. Both Ctrax and the neural network make a prediction for all frames in the video. 10% of the frames result in no overlap between neural network and Ctrax. Observation of these frames reveal that Ctrax has swapped the identity of the mouse with a sphere. Example frames show Neural Network predictions (red) and Ctrax predictions (blue).

Supplementary Table 1

Tracking Software Segmentation Algorithms

Tracking Software	Software Availability	Segmentation Approach (BGSLibrary notation)	Segmentation in BGSLibrary (Tested)
**Ctrax**	Open Source	Threshold with MOG2	Yes
**ToxTrac**	Open Source	Threshold with AGMM	Yes
**idTracker**	Open Source	Threshold with Temporal Mean BGS	Yes
**idTracker.ai**	Open Source	Threshold with Temporal Mean BGS	Yes
**CADABRA**	Open Source	Threshold with MOG2	Yes
**Ethovision**	Commercial	Threshold with Weighted Moving Mean	Yes
**MiceProfiler**	Open Source	Threshold with MOG2	Yes
**MOTR**	Open Source	Threshold with Temporal Mean BGS	Yes
**Cleversys TopScan**	Commercial	Threshold with Unspecified Gaussian Modelling Approach	NA
**Autotyping**	Open Source	Threshold with Temporal Mode BGS	Yes
**Automated Rodent Tracker**	Open Source	Custom BGS based on Canny Edges and Frame Difference	No
**Actimetrics Limelight**	Commercial	Threshold with Static Frame Difference	Yes

Supplementary Table 2

Training Parameters

Model	Parameter	Value
**All three**	Rotation Augmentation	±2.5 deg
	Translation Augmentation	±5.0 px
	Additive Noise Augmentation	μ=0.0, σ=5.0
	Brightness Augmentation	±5%
	Contrast Augmentation	±5%
	Optimizer	Adam
**Encoder-Decoder Segmentation Network**	Learning Rate	10^{-5}
	Batch Size	50
	Loss Segmentation	Softmax cross entropy
	Loss Cardinal Angle Prediction	Softmax cross entropy
**Regression Network**	Learning Rate	10^{-5}
	Batch Size	5
	Loss	Mean Squared Error
**Binned Classification Network**	Learning Rate	10^{-3}
	Batch Size	25
	Loss	Categorical cross entropy

Supplementary Table 3

Performance of trained networks on training data

Training	Black	Gray	Piebald	Albino	Difficult OFA	24Hr	KOMP2	Average
**Annotated Frame Count**	**8084**	**1739**	**0**	**5683**	**728**	**2099**	**1000**	
**Full Model Center Location Error, px**	0.72	0.85		1.44	1.69	1.83	1.38	1.13
**No Difficult Frames Model Center Location Error, px**	0.76	0.84		1.29	15.7*	2.35	1.83	1.71
**OFA Only Model Center Location Error, px**	0.73	0.77		1.21				0.91
**24 Hr Only Model Center Location Error, px**						2.71		
**KOMP2 Only Model Center Location Error, px**							1.06	

* indicates the model was not trained on this data, but inferred for comparison with other approaches.

Supplementary Table 4

Performance of trained networks on validation data

Validation	Black	Gray	Piebald	Albino	Difficult OFA	24Hr	KOMP2	Average
**Annotated Frame Count**	**278**	**63**	**0**	**196**	**31**	**93**	**83**	
**Full Model Center Location Error, px**	0.73	0.94		1.22	2.05	1.34	1.75	1.12
**No Difficult Frames Model Center Location Error, px**	0.72	0.92		1.26	26.7*	2.40	4.03	2.54
**OFA Only Model Center Location Error, px**	0.69	0.83		1.22				0.90
**24 Hr Only Model Center Location Error, px**						1.60		
**KOMP2 Only Model Center Location Error, px**							1.36	

* indicates the model was not trained on this data, but inferred for comparison with other approaches.

Supplementary Table 5

Performance comparison on test videos

Test	Black	Gray	Piebald	Albino	24Hr	KOMP2
**Annotated Frame Count**	**1174**	**1174**	**1174**	**1174**	**1195**	**1174**
**Full Model Center Location Error, px**	0.69	0.97	2.83	1.13	1.02	1.78
**OFA Only Model Center Location Error, px**	0.63	0.91	1.43	1.14		
**24Hr Only Model Center Location Error, px**					2.33	
**KOMP2 Only Model Center Location Error, px**						1.52
**Ctrax Center Location Error, px**	1.80	1.84	3.07	3.14	2.86	3.92

Supplementary Note 1

Fitting an Ellipse to a Mask

The same ellipse-fit algorithm was used as described in supplemental section 4.4.2 of the Ctrax paper[1]. While the paper uses a weighted sample mean and variance for these calculations, the segmentation neural network retains invariance to the situations in which they describe improvements. Additionally, we observe no difference between using weighted and unweighted sample means and variances.

Given a segmentation mask, the sample mean of pixel locations is calculated to represent the center position.

$$\mu_{x,y} = \frac{1}{N}\sum_i^N p_i \qquad (1)$$

Similarly, the sample variance of pixel locations is calculated to represent the major axis length (a), minor axis length (b), and angle (θ).

$$\sigma = \frac{1}{N}\sum_i^N \left(p - \mu_{x,y}\right)\left(p - \mu_{x,y}\right)^T \qquad (2)$$

To obtain the axis lengths and angle, an eigenvalue decomposition equation must be solved.

$$\sigma = U^T D U, \quad U = \begin{pmatrix} cos\theta & sin\theta \\ -sin\theta & cos\theta \end{pmatrix}, \quad D = \begin{pmatrix} \frac{a}{2} & 0 \\ 0 & \frac{b}{2} \end{pmatrix}^2 \qquad (3)$$

$$a = 2\sqrt{D_{11}}, \quad b = 2\sqrt{D_{22}}, \quad \theta = \mathrm{atan}(U_{12}, U_{21}) \qquad (4)$$

Supplementary Note 2

Annotated Datasets

We created 3 annotated datasets for training neural networks, each including a reference frame (input), segmentation mask, and ellipse-fit. Each dataset was generated to track mice in a different environment. An additional model was trained on all annotated examples for comparison. The exact number of frames represented in each dataset split as well as model performance can be found in Supplementary Tables 3 and 4.

The first annotated dataset uses images sampled from our standard open field arena video experiment and contains 16,802 annotated frames. This dataset was randomly split into a training set size of 16,234 frames and a validation set of 568 frames. The first 16,000 annotated frames were selected at random from 65 separate videos acquired from one of 24 testing arenas. We trained a model and found a small fraction of tracking issues when applying this model on the 1845 strain survey videos (0.007% of frames). We define tracking issues as the following: no mouse identified in the arena (eq 5), or a mouse becomes much larger the median size during an individual video (eq 6).

$$x = -1, y = -1, a = -1, b = -1 \qquad (5)$$

$$b_i > 4 * Median(b) \qquad (6)$$

An additional 802 frames across 50 new videos that perform poorly were identified, correctly annotated, and incorporated into the annotated dataset. The addition of these frames corrected the remainder 0.007% of frames in the strain survey.

The second annotated dataset uses images sampled from our 24-hour experiment, which uses the standard open field arena with ALPHA-dri bedding and a food cup under two distinct lighting conditions (day visible illumination and

night infrared illumination). For the dataset from this environment, we annotated a total of 2,192 frames across 6 videos of 4 day duration. Of the total number of annotated frames, 916 were taken from night illumination and 1,276 from the daylight illumination.

The third annotated dataset uses images sampled from the Accuscan Versamax Activity Monitoring Cages for the KOMP2 experiment. The dataset for this environment comprised 1,083 annotated frames. These annotations were all sampled across different videos (1 frame labeled per video) and 8 different arenas.

Supplementary Note 3

Statistic Reporting

Center Hypotenuse Prediction Error

We apply a log10 transformation of the data from independent images (n samples) to achieve a normal-like distribution. For mean comparison, we use a paired-end t-test. For variance comparisons, we use a paired-end F-test.

Dataset	n	t-test p-value	t-test 95% confidence interval	F-test p-value	F-test 95% confidence interval
Black	1174	<2.2e-16	0.507 – 0.557	<2.2e-16	0.385 – 0.484
Grey	1174	<2.2e-16	0.327 – 0.373	<2.2e-16	0.533 – 0.670
Piebald	1174	<2.2e-16	0.384 – 0.421	<2.2e-16	0.356 – 0.448
Albino	1174	<2.2e-16	0.460 – 0.503	5.285e-13	0.584 – 0.735
24-Hour	1195	<2.2e-16	0.090 – 0.111	3.212e-6	0.682 – 0.855
KOMP2	1174	<2.2e-16	0.310 – 0.363	2.631e-5	1.140 – 1.434

Supplementary References

1 Branson, K., Robie, A. A., Bender, J., Perona, P. & Dickinson, M. H. High-throughput ethomics in large groups of Drosophila. *Nature methods* **6**, 451 (2009).
