# Supplementary material for: Robust mouse tracking in complex environments using neural networks
Source: Commun Biol. 2019 Mar 29;2:124. doi: 10.1038/s42003-019-0362-1 (PMC6440983; doi:10.1038/s42003-019-0362-1)
Supplement: Supplementary file 2 — Description of Additional Supplementary Files [file 42003_2019_362_MOESM2_ESM.pdf]

Supplementary Movie 1

Supplemental_Movie_1.mp4

A comparison of mouse tracking across a variety of coat colors and environments using both our proposed encoder-decoder segmentation neural network (red) and Ctrax (blue). (0-22s) Black mice and (22-45s) grey mice in a white environment have strong agreement across approaches. When rearing on the wall, Ctrax starts to no properly fit the ellipse. (45-66s) Piebald mice in a white environment have good tracking, but depending upon the unique coat pattern may have incorrect shape predicted by Ctrax. (66-90s) Albino mice on a white background are a difficult problem for background subtraction approaches (Ctrax), while a neural network approach tracks appropriately. (90-112s) Black mice in the 24-hr setup, which contains bedding and a food cup, are difficult to create adequate background models for background subtraction approaches. A neural network approach learns to handle this difficulty. (112-134s) Black mice in the KOMP2 arena, which has reflective floors and walls, poses a difficult situation for background subtraction approaches. A neural network approach learns to not include reflections. Playback for clips in this video are at half-speed to better observe and compare tracking performance.

Supplementary Movie 2

Supplemental_Movie_2.mp4

A 1-minute sample from the two off-diagonal KOMP2 videos. In the first clip (0-62s), we observe a high degree of waddle in the gait as well as odd stride frequency. In the second clip (62-125s), we observe a hunched posture during locomotion as well as frequent sideways motion. Red ellipse denotes our neural network tracker prediction.

Supplementary Movie 3

Supplemental_Movie_3.mp4

A comparison of mouse tracking for an albino mouse in a white environment using our proposed encoder-decoder segmentation neural network (red) and Limelight (orange).

Supplementary Movie 4

Supplemental_Movie_4.mp4

A 1-minute sample from a 10-minute video with non-constant lighting conditions. Lights were manually adjusted during the duration of the test. Our proposed encoder-decoder segmentation neural network (red ellipse) is tolerant to changing light intensities while Ctrax (green ellipse) is only able to localize the mouse for about 50% of the frames.

Supplementary Movie 5

Supplemental_Movie_5.mp4

A 1-minute sample video from a video of a gray mouse with 3 large reflective move-able spheres placed in the arena. The spheres were agitated by slightly tipping the arena at 5s. Our proposed encoder-decoder segmentation neural network (red ellipse) functions well despite the large moving objects. Ctrax (green ellipse) does not have the capability of distinguishing between types of moving objects and jumps between correct prediction on the mouse and incorrect predictions on parts of the sphere.
