# Supplementary material for: Robust mouse tracking in complex environments using neural networks
Source: Commun Biol. 2019 Mar 29;2:124. doi: 10.1038/s42003-019-0362-1 (PMC6440983; doi:10.1038/s42003-019-0362-1)
Supplement: Supplementary file 3 — Reporting Summary [file 42003_2019_362_MOESM3_ESM.pdf]

nature research

Corresponding author(s): Vivek Kumar

Last updated by author(s): Feb 26, 2019

Reporting Summary

Nature Research wishes to improve the reproducibility of the work that we publish. This form provides structure for consistency and transparency in reporting. For further information on Nature Research policies, see Authors & Referees and the Editorial Policy Checklist.

Statistics

For all statistical analyses, confirm that the following items are present in the figure legend, table legend, main text, or Methods section.

n/a	Confirmed	
☐	☒	The exact sample size (*n*) for each experimental group/condition, given as a discrete number and unit of measurement
☐	☒	A statement on whether measurements were taken from distinct samples or whether the same sample was measured repeatedly
☐	☒	The statistical test(s) used AND whether they are one- or two-sided<br>*Only common tests should be described solely by name; describe more complex techniques in the Methods section.*
☐	☒	A description of all covariates tested
☐	☒	A description of any assumptions or corrections, such as tests of normality and adjustment for multiple comparisons
☐	☒	A full description of the statistical parameters including central tendency (e.g. means) or other basic estimates (e.g. regression coefficient) AND variation (e.g. standard deviation) or associated estimates of uncertainty (e.g. confidence intervals)
☐	☒	For null hypothesis testing, the test statistic (e.g. *F*, *t*, *r*) with confidence intervals, effect sizes, degrees of freedom and *P* value noted<br>*Give P values as exact values whenever suitable.*
☒	☐	For Bayesian analysis, information on the choice of priors and Markov chain Monte Carlo settings
☒	☐	For hierarchical and complex designs, identification of the appropriate level for tests and full reporting of outcomes
☐	☒	Estimates of effect sizes (e.g. Cohen's *d*, Pearson's *r*), indicating how they were calculated

Our web collection on statistics for biologists contains articles on many of the points above.

Software and code

Policy information about availability of computer code

Data collection	Video data for strain survey was collected using Actimetrics Limelight (http://www.actimetrics.com/products/limelight/) version 4103.<br>Video data for 24hr and KOMP2 was collected using custom code.<br>KOMP beam break data was collected using Versamax version 4.2.
Data analysis	All data analysis was conducted using custom code.<br>Neural network tracking code has been released publicly under an MIT license.<br>Statistical testing code was conducted in R.

For manuscripts utilizing custom algorithms or software that are central to the research but not yet described in published literature, software must be made available to editors/reviewers. We strongly encourage code deposition in a community repository (e.g. GitHub). See the Nature Research guidelines for submitting code & software for further information.

Data

Policy information about availability of data

All manuscripts must include a data availability statement. This statement should provide the following information, where applicable:

- Accession codes, unique identifiers, or web links for publicly available datasets
- A list of figures that have associated raw data
- A description of any restrictions on data availability

Neural network training sets used in this study are available on the kumarlab website https://www.kumarlab.org/2019/02/12/single-mouse-tracking-annotated-dataset/.
Pretrained neural networks used in this study are available on the kumarlab website https://www.kumarlab.org/2019/02/12/pre-trained-single-mouse-tracking-neural-network-models/.
Figure 2g and h's annotated frames are available on the kumarlab website as an annotated dataset.
Figure 3a contains strain survey distance traveled data and is available in MPD.

Field-specific reporting

Please select the one below that is the best fit for your research. If you are not sure, read the appropriate sections before making your selection.

☒ Life sciences ☐ Behavioural & social sciences ☐ Ecological, evolutionary & environmental sciences

For a reference copy of the document with all sections, see nature.com/documents/nr-reporting-summary-flat.pdf

Life sciences study design

All studies must disclose on these points even when the disclosure is negative.

Sample size	Sample sizes of 8 males and 8 females were used based on power analysis and conventional practice in the mouse neurogenetics field.
Data exclusions	Data were excluded if they were outliers (1.5 standard deviation from IQR).
Replication	Most behavioral tests were performed on multiple batches of animals.
Randomization	NA
Blinding	NA

Reporting for specific materials, systems and methods

We require information from authors about some types of materials, experimental systems and methods used in many studies. Here, indicate whether each material, system or method listed is relevant to your study. If you are not sure if a list item applies to your research, read the appropriate section before selecting a response.

Materials & experimental systems

n/a	Involved in the study	
☒	☐	Antibodies
☒	☐	Eukaryotic cell lines
☒	☐	Palaeontology
☐	☒	Animals and other organisms
☒	☐	Human research participants
☒	☐	Clinical data

Methods

n/a	Involved in the study	
☒	☐	ChIP-seq
☒	☐	Flow cytometry
☒	☐	MRI-based neuroimaging

Animals and other organisms

Policy information about studies involving animals; ARRIVE guidelines recommended for reporting animal research

Laboratory animals	All strains and numbers are stated in the figures, figure caption, or methods.<br>All animals used were ordered directly from the JAX colonies. Mice were housed in barrier specific pathogen free (SPF) conditions with Light:Dark (LD 12:12) according to The Jackson Laboratory Institutional Animal Care and Use Committee guidelines. Mice were weighed and allowed to acclimate in the testing room for 30-45 minutes before the start of video recording. Where available, 8 males and 8 females were tested from each inbred strain and F1 isogenic strain.
Wild animals	NA
Field-collected samples	NA
Ethics oversight	Animals were tested in accordance with approved protocols from The Jackson Laboratory Institutional Animal Care and Use Committee guideline.

Note that full information on the approval of the study protocol must also be provided in the manuscript.
